# Barley C-Hordein as the Calibrant for Wheat Gluten Quantification

**DOI:** 10.3390/foods9111637

**Published:** 2020-11-10

**Authors:** Xin Huang, Kaiyue Ma, Sara Leinonen, Tuula Sontag-Strohm

**Affiliations:** Department of Food and Nutrition, Faculty of Agriculture and Forestry, University of Helsinki, Agnes Sjöberginkatu 2, PL66, FI-00014 Helsinki, Finland; kaiyue.ma@helsinki.fi (K.M.); sara.leinonen@paulig.com (S.L.); tuula.sontag-strohm@helsinki.fi (T.S.-S.)

**Keywords:** R5, ELISA, gluten-free, contamination, reference material, oats

## Abstract

The lack of certified reference materials has been one major challenge for gluten quantification in gluten-free products. In this study, the feasibility of using barley C-hordein as the calibrant for wheat gluten in R5 sandwich enzyme-linked immunosorbent assay (ELISA) was investigated. The gluten composition and total gluten R5 reactivity ranged largely depending on the genotypes and the growing environment. The conversion factor of gliadin to gluten averaged 1.31 for common wheat, which is smaller than the theoretical factor of 2. Each gluten group had varying reactivity against the R5 antibody, where ω1.2-, γ- and α-gliadins were the main reactive groups from wheat gluten. A mixture of wheat cultivars or one single cultivar as the reference material can be difficult to keep current. Based on the average R5 reactivity of total gluten from the 27 common wheat cultivars, here we proposed 10% C-hordein mixed with an inert protein as the calibrant for wheat gluten quantification. In spiking tests of gluten-free oat flour and biscuits, calibration using 10% C-hordein achieved the same recovery as the gliadin standard with its cultivar-specific conversion factor. For its good solubility and good affinity to the R5 antibody, the application of C-hordein increases the probability of developing a series of reference materials for various food matrices.

## 1. Introduction

Consumption of gluten-containing products is involved in several immune disorders, including celiac disease, wheat allergy and wheat sensitivity. Celiac disease is an autoimmune disorder triggered by ingestion of gluten proteins from Triticeae, and causes small intestine mucosa damage [1]. Gluten proteins are referred to as seed storage prolamin proteins; in wheat they are called gliadins and glutenins, in barley they are called hordeins, and in rye they are called secalins [2]. To maintain a healthy condition, celiac patients must practice strict avoidance and keep a gluten-free diet. Therefore, residual gluten or cross contamination in products needs to be quantified correctly. The Codex Alimentarius standard 118-1979 (2008) [3] defined the current threshold for gluten-free products as 20 mg/kg, and the European Union [4], the United States of America [5] and Canada [6] also adopted the same threshold. The most commonly used quantification method is the enzyme-linked immunosorbent assay (ELISA) method and several commercial kits based on different gluten antibodies are available on the market, including R5, G12, A1, and Skeritt antibodies. The R5 Mendez based ELISA method is the type I method suggested by the Codex Alimentarius standard. The R5 antibody is a monoclonal antibody raised against rye secalins and mainly recognises the epitope QQPFP [7]. Ideally, in a sandwich type R5 ELISA designed for intact protein detection, the R5 antibody detects gliadin fractions from a wheat sample extracted from a food matrix. The measured gliadin content is calibrated against a gliadin standard, and finally total gluten content of the sample is calculated from gliadin concentration with a conversion factor 2 based on the theoretic ratio (1:1) of gliadins:glutenins. Because of the cross reaction of the R5 antibody with other non-wheat Triticeae prolamins, the assay is also used for detection of barley and rye prolamins, because usually the contamination source is unknown from a sample. However, the quantification of barley prolamin using a wheat gliadin standard caused about five times overestimation in R5 sandwich ELISA [8,9,10]. A separate barley standard was needed for calibration, thus in our previous study [10], we isolated barley C-hordein and the calibration with C-hordein achieved more accurate quantitation than the gliadin standard when determining the barley contamination in gluten-free oats. The primary structure of C-hordein, much like the homologues in wheat and rye (ω-gliadins and ω-secalins, respectively) consists almost entirely of repeats of the QQPFP motif, which is the main recognition sequence for the R5 antibody [2]. C-hordein from three selected barley cultivars with different RP-HPLC patterns had equal and good R5 recognition. To calibrate the total hordein amount, isolated C-hordein was mixed with an inert protein in proportion (40%) to represent the reactivity of total hordein against the R5 antibody [10]. Thus, the approach of a reference material using one group of gluten protein was first proposed.

There are several challenges in gluten detection by ELISA methods that have been critically discussed, including the extraction methods, the antibody specificity and detection, and the calibration step with the reference material [11,12]. Using a generic extraction method and a common calibrator, a study revealed that the comparability of all gluten detection commercial kits was very limited [13]. The lack of a certified reference material has been one major challenge. Several gluten detection kits use gluten or gliadin isolate as the reference material for their calibrations. Of these, the Prolamin working group (PWG) gliadin standard, made with a mixture of 28 cultivars from the UK, Germany and France from 1999, was the best characterised. The PWG gliadin standard consisted of a solution with a total protein content of 96.7%, of which the gliadin content was 86.4%. Of this, the α/β-gliadins comprised 41.7%, γ-gliadins 47.0%, ω1,2-gliadin 6.3% and ω5-gliadin 5.0% [14]. However, this standard was not accepted as certified reference material as it did not have sufficient purity and it was not reproducible [11]. Additionally, none of the wheat cultivars used are currently important on the market and the stock of this batch of standard material will run out soon. This brings up the issue of the development of new reference materials for wheat gluten quantification and for other cereals. The strategies proposed are the use of whole wheat flour, gliadin or gluten isolate from a mixture of cultivars or from one single cultivar, incurred matrix, or a single protein [11,15,16]. Based on these strategies and led by the PWG, five cultivars were selected after characterisation as the basis for the development of a new reference material [15,16]. The gluten composition of these five cultivar flours varied between harvest years, but a blend of the five cultivars overcame this variability and showed advantages over use of a single cultivar in ELISA responses [17,18]. A gluten isolate or a gliadin isolate from the blend of the five cultivars showed the same protein composition as the native flour [18]. The varying gluten composition by genotype and environmental factors certainly increased the difficulty of satisfying the criteria of reference material. A series of reference materials that are suitable for different cereals and food matrices is required for reliable gluten quantification.

The aim of this study was to investigate the feasibility of a single protein group, barley C-hordein, for calibration of wheat gluten in R5 ELISA assay. We collected 27 common wheat cultivars that are important in the recent market and investigated their gluten compositions and their total gluten reactivity against the R5 antibody. Based on their R5 reactivities, we proposed the use of barley 10% C-hordein for the calibration of wheat gluten. To evaluate the calibration in raw and heat-treated foods, three wheat cultivars, with varying protein composition and R5 reactivities, were selected and spiked in gluten-free oat flour and oat biscuits made from the spiked flour, respectively.

## 2. Materials and Methods

### 2.1. Materials

Based on production in 2016–2017, samples of 27 high-yielding common wheat cultivars (*Triticum aestivum* L.) were collected from seven countries, including Anniina, Quarna and Amaretto from Finland; Julius, Brons, and Hereford from Sweden; Julius, Kerubino, and Patras from Germany; Cellule and Apache from France; Siskin, Lili, Crusoe, Zulu, Claire, Revelation, and Britannia from the UK; Brandon, Steller, Foremost, and Penhold from Canada; Gregory, Lancer, Spitfire, Suntop, and Mace from Australia. Hull-less barley (*Hordeum vulgare*) cultivar Jorma was obtained from Villala, Finland. All chemicals were analytical grade.

### 2.2. Gluten Composition Analysis by Reverse-Phase-High Performance Liquid Chromatography (RP-HPLC)

The grain seeds were milled with a sample mill (Koneteollisuus Oy, KT-30, Klaukkala, Finland). The protein extraction method was slightly modified following modified Osborne sequential extraction [19], 100 mg flour was extracted by 1 mL 0.4 M NaCl + 0.067 M HKNaPO_4_ (pH 7.6) at room temperature (20–23 °C) for 10 min for two times. These two extractions were combined to form the Albumin + Globulin (Alb + Glo) fraction. The gliadin fraction was then extracted with 0.5 mL 50% (*v/v*) propan-1-ol at 60 °C for 10 min three times and combined [20]. The glutenin fraction was extracted in 1 mL of 50% (*v/v*) propan-1-ol with 5% (*v/v*) β-mercaptoethanol at 60 °C twice and the extracts were combined. Duplicate samples of these three protein fractions were obtained from each cultivar. After filtration through a 0.45 µm GHP membrane (Pall Corporation, Ann Arbor, MI, USA), these fractions were analysed by RP-HPLC using the Agilent Technologies 1200 series system with a diode array detector (Agilent, Santa Clara, CA, USA). Protein solutions were separated at 50 °C on a SUPELCO Discovery Bio Wide Pore C8, 5 µm, 25 cm × 4.6 mm (Sigma-Aldrich, St.Louis, MO, USA) with matching guard column 2 cm × 4 mm on a gradient of 2 min, 0% B; 4 min, 24% B; 52 min, 56%; 58 min, 90% B; 65 min, 0% B, where buffer B consisted of acetonitrile with 0.1% (*v/v*) trifluoroacetic acid and buffer A consisted of 0.1% (*v/v*) trifluoroacetic acid in mQ water. The injection volume for Alb + Glo fraction was 50 µL, for gliadin fraction it was 25 µL and for glutenin fraction it was 50 µL. UV detection was set to 210 nm. Protein content was calculated based on the peak area using bovine serum albumin (BSA) as the standard in the linear range (0–80 µg). Cultivar-specific conversion factors were calculated as its total gluten proportion divided by its total gliadin proportion.

### 2.3. Isolation of Total Gluten and Their R5 Reactivity

To evaluate the total gluten R5 reactivity, gluten isolates of the 27 wheat cultivars were prepared by modified Osborne fractionation. Albumins and globulins were removed by extraction three times with the buffer described in Section 2.2, total gluten of each cultivar was extracted from 5 g flour by 30 mL 50% (*v/v*) propan-1-ol with 5% (*v/v*) β-mercaptoethanol at 60 °C for 30 min twice. After centrifugation (18,000× *g*) for 10 min, the supernatant was collected and dialysed (SnakeSkin Dialysis Tubing, 3.5K MWCO, ThermoFisher, Rockford, IL, USA) against mQ-H_2_O with at least three changes. The supernatant was then lyophilized and the nitrogen content was determined by the Dumas combustion method (VarioMax CN, Elementar Analysensysteme GmbH, Langenselbold, Germany) and multiplied by 5.7 to give the protein content [21]. The 0.25 g gluten isolate was dissolved in 2.5 mL patented cocktail solution (R7006) and gluten content was quantified using the Ridascreen Gliadin R7001 following kit instructions (R-Biopharm, Darmstadt, Germany). In order to calculate the EC50 value of the total gluten R5 reactivity, a series of dilutions of each gluten solution was prepared using at least six measuring points on the curve. The EC50 value indicates the half concentration of the maximal antibody binding and was calculated using a non-linear four parameter curve fit by Graphpad Prism 8 (San Diego, CA). A C-hordein isolate preparation has been described [10], briefly hordeins were extracted by aqueous alcohol solution and separated with an ion exchange chromatographic method, the C-hordein fraction was collected, dialyzed and lyophilized. A stock solution of C-hordein and BSA in 60% (*v/v*) ethanol were made at the same concentration, and then mixed at 10% (1 C-hordein: 9 BSA), 20% (2 C-hordein: 8 BSA) and 30% (3 C-hordein: 7 BSA) (*v/v*). The 10%, 20% and 30% C-hordein standard solutions were further diluted to fit into the ELISA reaction curve.

### 2.4. Isolation of Gluten Subunits and R5 Sandwich Responses

Based on retention time, fractions comprising ω5-gliadin, ω1,2-gliadin, α-gliadin, and γ-gliadin in the gliadin fraction, HMW-glutenin and low molecular weight (LMW)-glutenin in the glutenin fraction of cultivar Crusoe were collected from RP-HPLC separation [19]. The protein content was determined as before. The fractions were dried under nitrogen flow first, and then with vacuum by SpeedVac (Savant SC110A Concentrator, San Diego, CA, USA). The fractions were dissolved in 0.25 mL cocktail solution and then in 80% (*v/v*) ethanol, the gluten subunits were diluted into a suitable range for analysis in R5 sandwich ELISA (Ridascreen Gliadin R7001, R-Biopharm, Darmstadt, Germany). The fractions of ω1,2-gliadins of 5 cultivars (Amaretto, Anniina, Brandon, Claire and Lili), and the fractions of α-gliadins and γ-gliadins of 4 cultivars (Amaretto, Apache, Brandon and Foremost) with distinct HPLC patterns were also collected and analysed with sandwich R5 ELISA as before. A series of dilutions was prepared and the EC50 value was calculated as in 2.3.

### 2.5. Spiking Oat Flour and Oat Biscuits and Calibration with C-Hordein Standard

Wheat cultivars of high, medium and low total gluten R5 reactivity, Steller, Zulu and Apache, respectively, were selected for spiking tests to demonstrate the performance of the calibration when spiked with different cultivars. The three wheat flours were spiked into gluten-free oat flour (Provena, Raisio, Finland) at 1 g/kg (1000 mg/kg) concentration in three consecutive steps (10 × 10 × 10) for better homogeneity. Depending on the flour total protein content and gluten composition, the spiked gluten concentration was around 100 ppm. In order to investigate the effect of food processing/heat treatment on the ELISA analysis, oat biscuits were prepared by mixing eggs 20 g, sugar 50 g, and butter 50 g with 100 g of the spiked oat flour. The biscuits were baked in a conventional oven at 180 °C for 16 min. After cooling to room temperature, the loss of moisture was measured. The gluten content of the spiked oat flour and biscuits was measured following Ridascreen Gliadin R7001 ELISA. Negative control biscuits were made from the gluten-free oat flour to ensure no contamination occurred during the biscuit-making process. The gluten content was calculated by (1) calibration with the gliadin standard, then multiplied with the conversion factor 2; (2) calibration with gliadin standard and then multiplied with the cultivar-specific conversion factor obtained; and (3) calibration with 10% C-hordein standard and no conversion. The gluten protein recovery was determined by $\frac{calibration\;gluten\;content}{theoretical\;gluten\;content}\times 100\%$, where theoretical gluten content = nitrogen content × 5.7 × gluten proportion from HPLC. The spiked flour and biscuit samples were prepared in two biological replicates; for measurement of each biological replicate, two extraction replicates were made and measured in four technical replicates in ELISA and calculated with two dilution factors.

### 2.6. Statistical Analysis

The significance test of difference of gluten protein compositions results from RP-HPLC was conducted by SPSS 10.0, using one-way ANOVA analysis with Tukey’s HSD test; within each protein group the significance (*p* < 0.05) was indicated by different letters. The significance test of difference of protein recovery from the spiking test was determined by one-way ANOVA analysis and Tukey’s test by Graphpad Prism 8; the level of significance was indicated by asterisk (ns, not significant; * *p* < 0.05, ** *p* < 0.01, *** *p* < 0.001, **** *p* < 0.0001)

## 3. Results

### 3.1. Protein Composition of 27 Common Wheat Cultivars

**Figure 1:**
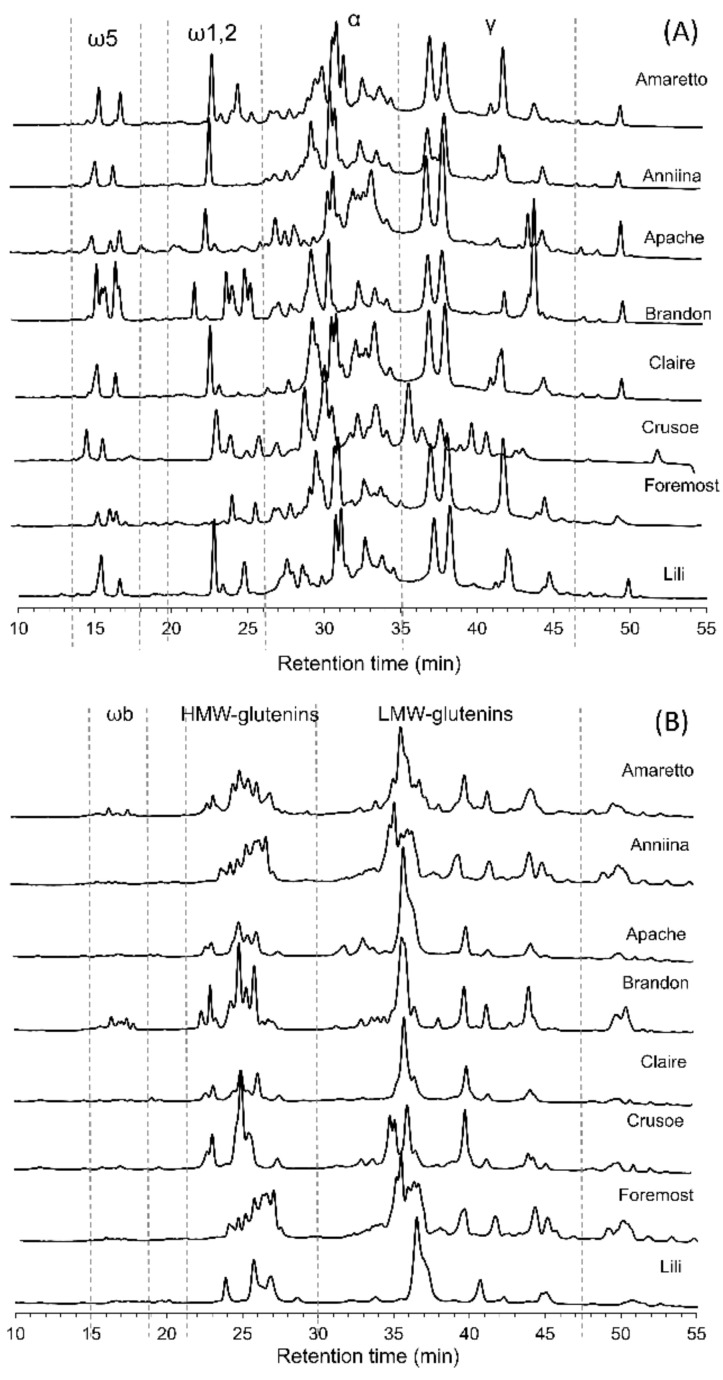
Chromatogram of gliadins fraction (Panel (**A**)) and glutenins fraction (Panel (**B**)): separation on a C8 column from cultivar Amaretto, Anniina, Apache, Brandon, Claire, Crusoe, Foremost and Lili at wavelength 210 nm. (ω5, ω5-gliadins; ω1,2, ω1,2-gliadins; α, α-gliadins; γ, γ-gliadins; ωb, glutenin-bound gliadins; high molecular weight (HMW)-glutenins, high molecular weight-glutenins; low molecular weight (LMW)-glutenins, low molecular weight-glutenins).

According to the hydrophobicity of the proteins, gliadin and glutenin fractions were separated using a C8 column. Based on the retention time, in the gliadin fraction the proteins were eluted in an order of ω5-gliadin, ω1,2-glidins, α-gliadins and γ-gliadins, while in the glutenin fraction the proteins were eluted in an order of glutenin-bound ω-gliadins, high molecular weight (HMW)-glutenins and low molecular weight (LMW)-glutenins [19]. Examples of prolamin separation of 8 cultivars are shown in Figure 1; albumins/globulins fraction were not shown here. Peaks of each protein/prolamin group were integrated and based on the peak area of each protein/prolamin group; the relative proportions of albumins/globulins, gliadins and glutenins varied widely in the 27 wheat cultivars (Table 1). Gluten proteins were the major proteins in wheat, comprising 81.5% of the total proteins on average, while albumins/globulins were the minor component comprising 18.3% of the total proteins on average. Gliadins were the major wheat proteins and comprised more than half in every cultivar except cv. Apache. The α-gliadins and γ-gliadins were the major gliadins comprising, on average, 30.0% and 23.8% of the total proteins, respectively. The ω-gliadins were the minor fraction of gliadins and comprised 8.3% of the total proteins on average. The proportion of glutenins was always lower than that of gliadins, and the proportion of HMW-glutenins was lower than that of the LMW-glutenins in all 27 cultivars. Based on the proportion, the cultivar specific conversion factor from gliadin to gluten for the 27 cultivars ranged from 1.19 to 1.48, but was always lower than the theoretical conversion factor of 2. Less than 1.3% of the protein was so-called glutenin-bound ω-gliadins. The proportions of Alb+Glo, ω5-gliadins, and γ-gliadins differed significantly in cv Julius depending on where it was grown: Germany or Sweden.

### 3.2. The R5 Reactivity of Total Gluten of 27 Cultivars

Total gluten of each cultivar was isolated and analysed in sandwich R5 ELISA. The R5 reactivity was summarised as its EC50 value (Table 2) by calculation from a non-linear four-parameter curve fit. The fitted curves and their 95% confidence intervals are presented in Figure S1, as standard deviation is not recommended for the EC50 value. The fitted curves gave acceptable EC50 estimates for total gluten R5 reactivity for most cultivars. The EC50 values of the total gluten of 27 cultivars varied greatly and ranged from the strongest R5 reactivity of 30.4 ng/mL for cv. Crusoe to the weakest R5 reactivity of 664.3 ng/mL for cv. Britannia (Table 2). The average total R5 reactivity of the 27 cultivars had an EC50 value of 62.1 ng/mL, which was closer to 10% (*v/v*) C-hordein with an EC50 value of 61.5 ng/mL (Figure S1).

### 3.3. Reactivity of Gluten Types Against R5 Antibody

To further investigate the R5 reactivity of each prolamin type, the gliadin and glutenin fractions of cv. Crusoe were analysed. The EC50 value of each prolamin type demonstrated widely varying R5 reactivities. The ω1.2-gliadins were the most reactive prolamin type, followed by γ-gliadins, α-gliadins and HMW-glutenins, while LMW-glutenins and ω5-gliadins had very limited reactivities (Table 3, Figure S2). Because the ω1.2-, α- and γ-gliadins were the main prolamins recognized by the R5 antibody, we further investigated whether the same prolamin type from different cultivars demonstrated different R5 reactivity. The RP-HPLC chromatogram of the gliadin fraction showed a distinct pattern of ω1,2-gliadins from cv. Amaretto, Anniina, Brandon, Claire and Lili (Figure 1) and their EC50 values of R5 reactivities ranged from 5.3 ng/mL to 14.0 ng/mL (Table 4, Figure S3). The ω1.2-gliadins of cv. Anniina exhibited a single peak and showed the highest R5 reactivity with an EC50 value 5.3 ng/mL. The chromatograms of α- and γ-gliadins were more complex, containing multiple peaks and peak shoulders. In order to demonstrate their varying activities in cultivars, α- and γ-gliadins from four cultivars were isolated and their EC50 value of α-gliadins ranged from 26.7 ng/mL to 41.6 ng/mL, and their EC50 value of γ-gliadins ranged from 17. ng/mL to 33.7 ng/mL.

### 3.4. Calibration of Gluten Content in Spiked Oat Flour and Oat Biscuit Samples

**Figure 2:**
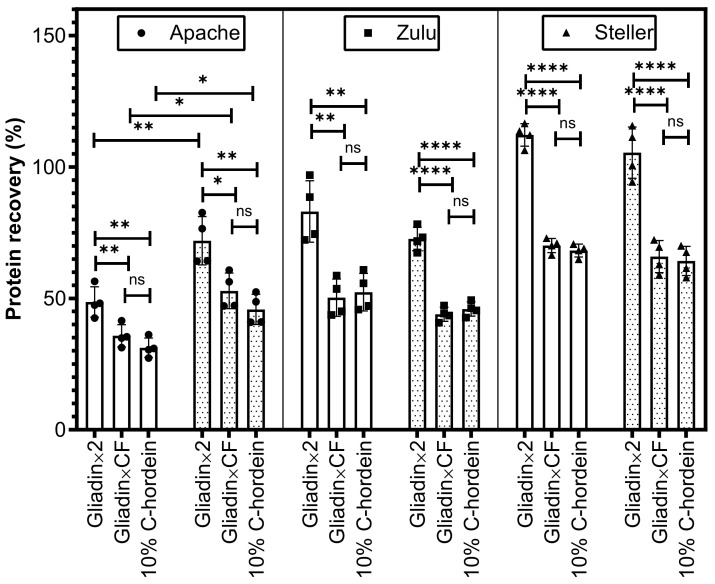
Protein recovery (%) of gluten in wheat spiked oat flour and biscuits measured by R5 ELISA. The recovery was calculated by measured gluten/theoretical gluten × 100%. Measured gluten was the calibration with gliadin and conversion factor 2, gliadin with cultivar-specific conversion factor (CF), and 10% C-hordein, respectively. Three common wheat cultivars Apache, Zulu and Steller were spiked in gluten-free oat flour, respectively (blank bars). The wheat-spiked oat flour was made into biscuits (dotted bars). Four measurement points indicated two biological replicates and two samples taken from each biological replicate. The error bar indicates their standard deviation. The significant test of variance between each calibration used one-way ANOVA with a Tukey correction (ns, not significant; * *p* < 0.05, ** *p* < 0.01, *** *p* < 0.001, **** *p* < 0.0001).

The wheat cultivars Apache, Zulu and Steller were chosen for spiking tests because these three cultivars had statistically significant different relative proportions of ω1.2-gliadins (2.1%, 3.6%, 7.5%, respectively), α-gliadins (21.9%, 37.1%, 27.4%, respectively) and γ-gliadins (20.7%, 23.0%, 28.9%, respectively) between cultivars. In addition, their total gluten R5 reactivity represented a high, medium and low level of EC50 value (376.6, 66.8, 30.7 ng/mL, respectively), of which cv. Zulu was close to the average of all 27 cultivars. This helps to understand the feasibility of the calibration in a real situation when an unknown cultivar is a contaminant. Details of the calculation and calibration steps are shown in Table 5 and Table 6. Depending on the protein content and gluten proportion, the theoretical gluten content spiked in oat flour ranged from 72.7 to 156.8 mg/kg, and the theoretical gluten content spike in the oat biscuits ranged from 37.7 to 80.4 mg/kg. In the calibration steps, calibration with gliadin, and with a conversion factor of 2, gave a significantly higher protein recovery in all flour and biscuit samples of the three cultivars, compared to the other two calibrations used. Calibration with 10% C-hordein gave a result that was not significantly different from the calibration with gliadin standard and its cultivar-specific conversion factor (Figure 2). The gluten protein recovery of the oat biscuits spiked from cv. Zulu and cv. Steller was only slightly lower (but not significantly) than the gluten protein recovery in oat flour. Interestingly, the gluten protein recovery of cv. Apache spiked oat biscuits increased significantly compared with the ones in oat flour with all three calibrations.

## 4. Discussion

This study investigated the prolamin compositions of the wheat cultivars on the current market and their total gluten reactivities towards the R5 antibody. The results showed that the gluten composition varied greatly among popular common wheat cultivars; gliadins were the main component and the ratio of gliadins to glutenins ranged from 2.07 to 5.34. Gliadins were also the main recognition for the R5 antibody, of which ω1.2-gliadins had the strongest reactivity. In addition, the same type of gliadins from different cultivars also varied in R5 reactivity. The complexity of gluten composition and the varying R5 reactivity of each gluten type explained the large range of total gluten R5 reactivity of these 27 cultivars. Thus, it would be challenging to establish and maintain a reference material based on a single cultivar or a mixture of wheat cultivars. The calibration with 10% C-hordein standard achieved the same protein recovery as the gliadin standard with cultivar-specific conversion factor in all three wheat cultivar spiked oat flour or biscuits.

The main recognition of R5 antibody is epitope QQPFP, the amount of which in prolamin sequences determines the varying reactivity. For example, eighteen repeats of QQPFP were found in ω1.2-gliadins (Uniprot entry D2KKB1), 3 repeats from γ-gliadins (P06659), 5 repeats from γ-gliadins (P21292), and 1 repeat from α-gliadins (P04721, P04723, P04725, P18573), explaining the main R5 reactivity of gluten from these prolamins [22]. Because of the complexity of wheat prolamins, this study showed the same prolamin group from different cultivars varied to some extent. No R5 epitope was found in ω5-gliadins (Q40215) and only one was found in LMW-glutenins (P13615); this accounted for their minimum reactivities. Although no R5 epitope was found in HMW-glutenin x-type (P10388), and y-type (P10387), they had some limited reactivity against the antibody. A similar phenomenon was observed in Western blot with the R5 antibody [20,23]. In addition, the R5 antibody also recognises homologous epitopes such as LQPFP, QLPYP, QQSFP, QQPYP and PQPFP [24]. The mild recognition of HMW-glutenins complicated total gluten quantitation when, theoretically, the R5 antibody detects only gliadins and therefore renders the conversion factor incorrect. The varying total gluten R5 reactivity can be partially explained by the proportion of ω1.2-gliadins. For example, cv. Steller and cv. Brandon contained 7.5% and 9.0% of ω1.2-gliadins in wheat proteins, respectively, and also had strong total gluten R5 reactivity with EC50 values of 30.7 ng/mL and 35 ng/mL, respectively. On the other hand, cv. Brons and cv. Apache had only 1.9% and 2.0% ω1.2-gliadins, respectively, and consequently had weak total gluten R5 reactivity with EC50 values of 325.6 ng/mL and 376.6 ng/mL, respectively. However, a good correlation of the EC50 and ω1.2-gliadins cannot be established (Figure S4). Wheat cultivar Claire had a low ω1.2-gliadins proportion of 2.8% but a strong total gluten R5 reactivity with an EC50 value of 38.6 ng/mL. This may be explained by a high ratio of gliadins to glutenin (4.24), such that α- and γ-gliadins contributed mostly to the total gluten R5 reactivity.

In this study, the ratio of gliadins to glutenins of 27 wheat cultivars ranged from 2.07 to 5.34 based on Osborne sequential extraction. In previous studies, with similar RP-HPLC methods, the ratio ranged from 1.51 to 3.14 (54 cultivars) [25], 1.7 to 4.2 (13 cultivars) [26], 2.0 to 4.2 (23 cultivars) [16], and 1.93 to 3.10 (5 cultivars) [15]. These data suggest that the conversion factor 2, recommended by the Codex standard 118-1979, is higher than the actual conversion factor for common wheat (1.19–1.48 this study, 1.24 to 1.50 [16], 1.32–1.66 [25]). One must be aware that the Osborne sequential extraction based on solubility cannot provide clear-cut classification of wheat proteins, because of the fact that some of the glutenins or albumin/globulins co-extracted into gliadin fraction or even co-eluted in the RP-HPLC. For example, amylase/trypsin inhibitors were found to be co-extracted in the gliadin fraction and co-eluted in the ω-gliadins fraction in RP-HPLC [27,28,29].

Due to varying compositions of wheat cultivars and varying R5 reactivities of gluten types, the total gluten R5 reactivities of these cultivars resulted in a large range. C-hordeins, similar to ω1.2-gliadins, have good binding to the R5 antibody and were selected as the base of the reference material, for their consistently higher proportion of barley and their relative ease of preparative isolation. Ideally, a reference material would comprise a total gluten isolate, but its solubility and stability is poor in a solution due to the aggregative nature of gluten proteins. Thus, a gliadin standard was developed because gliadins are monomeric and soluble in aqueous alcohol solution. The R5 antibody detected epitopes related to QQPFP and C-hordein is a good source of QQPFP epitopes. Interestingly, although three spiked wheat flours had varying gluten composition and R5 reactivity, in both raw and cooked foods, calibration with 10% C-hordein achieved comparable results as the gliadin standard with the cultivar-specific conversion factors. The reason might be that calibration with 10% C-hordein represents the average of total gluten. The calibration with the gliadin standard represents only one part of gluten and therefore to achieve correct quantitation of total gluten, a cultivar-specific conversion factor is needed. However, this is not normally possible as the contaminant source/cultivar is unknown.

Using a conversion factor of 2 achieved higher protein recovery compared to the theoretical gluten content. This raises a question concerning the efficiency of the two-step extraction procedure, firstly with the “cocktail solution” containing guanidine hydrochloride and 2-mercaptoethanol, and secondly with an aqueous alcohol solution. This procedure improved the gliadin extractability for unheated and heated food compared to the conventional extraction method with 60% aqueous alcohol [30]. Another extraction buffer with reducing agent tris(2-carboxyethyl)phosphine (TCEP) achieved similar recovery [31,32]. In those tests the spike was isolated gliadin, of which the extractability is higher than that of whole flour [33]. In a spiked flour test, the gliadin recovery determined by the R5 and G12 ELISA assay against RP-HPLC results of gluten-free flour ranged from 72.8% to 162.5% and its cookies ranged from 49.1% to 192.0% [15]. In a comparison study of ELISA kits, the 2-step extraction procedure of vital gluten as in R-Biopharm, Agraquant or Transia showed, on average, a lower extraction efficiency than a Japanese official allergen detection extraction method [13]. A higher conversion factor of 2, in a way, compensated for the incomprehensive nature of the two-step extraction procedure. A better extraction procedure is needed to improve the extractability of gluten proteins, such as the introduction of a prolonged extraction time or a multi-step extraction.

## 5. Conclusions

This study investigated the complexity of wheat protein composition by RP-HPLC, and wheat prolamins reactivity towards R5 ELISA. A reference material that can represent the total gluten reactivity gives a more reliable result, such as 10% C-hordein used in this study, rather than calibration with gliadin, with a conversion factor of 2. For the development of a new reference material for wheat, C-hordein is a candidate on account of its high affinity to the R5 antibody and good solubility in aqueous alcohol. Following our previous study of using barley C-hordein (40%) as the reference material for barley prolamin calibration, this study proposes C-hordein (10%) as the reference material for wheat gluten detection in R5 antibody-based ELISA.

## Figures and Tables

**Table 1:** Protein composition of 27 common wheat cultivar flours. (ωb-gliadins, glutenin-bound ω-gliadins; HMW, high molecular weight; LMW, high molecular weight).

Cultivar	Albumins + Globulins (%)	ω5-Gliadins (%)	ω1,2-Gliadins (%)	α-Gliadins (%)	γ-Gliadins (%)	ωb-Gliadins (%)	HMW-Glutenins (%)	LMW-Glutenins (%)	Total Gliadins (%)	Total Glutenins (%)	Gliadins: Glutenins	Conversion Factor (Gluten:Gliadins)
Amaretto	17.7 ^edf^	3.3 ^abcdef^	5.1 ^jk^	25.1 ^b^	21.0 ^abcd^	1.0 ^ijk^	9.6 ^j^	17.2 ^n^	55.4 ^b^	26.8 ^p^	2.07	1.48
Anniina	15.6 ^bcde^	4.1 ^ghij^	2.8 ^cd^	33.0 ^jk^	24.3 ^fghi^	0.7 ^efghij^	6.2 ^efgh^	13.2 ^fghi^	64.9 ^hij^	19.3 ^ghijkl^	3.35	1.30
Apache	28.7 ^i^	3.0 ^abcd^	2.1 ^ab^	21.9 ^a^	20.7 ^abc^	0.8 ^fghij^	6.0 ^defgh^	16.7 ^lmn^	48.5 ^a^	22.7 ^mno^	2.14	1.47
Brandon	11.9 ^ab^	8.9 ^m^	9.0 ^m^	28.4 ^cde^	26.6 ^jk^	0.4 ^abcd^	6.4 ^fgh^	8.3 ^ab^	73.2 ^m^	14.7 ^bc^	4.98	1.20
Britannia	23.7 ^gh^	3.7 ^defgh^	4.0 ^fgh^	30.9 ^fghi^	25.4 ^hijk^	0.3 ^ab^	4.3 ^a^	7.8 ^a^	64.1 ^ghi^	12.0 ^a^	5.34	1.19
Brons	25.0 ^hi^	3.8 ^efgh^	1.9 ^a^	29.6 ^efg^	20.2 ^ab^	0.9 ^hij^	4.8 ^abc^	13.5 ^ghij^	56.5 ^bc^	18.3 ^fghijk^	3.08	1.32
Cellule	13.7 ^abc^	7.8 ^l^	4.8 ^ij^	31.5 ^ghij^	19.0 ^a^	1.3 ^k^	6.7 ^ghi^	15.0 ^ijkl^	64.4 ^hij^	21.7 ^lmn^	2.97	1.34
Claire	25.2 ^hi^	3.2 ^abcde^	2.8 ^bcd^	30.8 ^fghi^	23.6 ^efgh^	0.1 ^a^	4.3 ^a^	10.0 ^bcd^	60.4 ^defg^	14.3 ^ab^	4.24	1.24
Crusoe	17.5 ^cdef^	3.0 ^abcd^	4.8 ^ij^	34.5 ^k^	21.0 ^abcd^	0.6 ^bcdefg^	7.1 ^hi^	11.5 ^def^	63.9 ^ghi^	18.5 ^fghijk^	3.45	1.29
Foremost	15.1 ^bcd^	2.8 ^abc^	2.9 ^cd^	31.8 ^hij^	29.2 ^l^	0.3 ^abc^	5.2 ^abcde^	12.5 ^efgh^	67.0 ^ijk^	17.7 ^efghi^	3.78	1.26
Gregory	18.1 ^def^	4.0 ^fghi^	3.3 ^def^	27.0 ^bc^	22.4 ^bcdef^	0.5 ^bcde^	7.8 ^i^	16.9 ^mn^	57.1 ^bcd^	24.7 ^op^	2.31	1.43
Hereford	25.2 ^hi^	3.2 ^abcde^	2.4 ^abc^	29.6 ^efg^	23.6 ^efgh^	0.7 ^defghi^	4.9 ^abcd^	10.3 ^cd^	59.4 ^cdef^	15.2 ^bcd^	3.90	1.26
Julius DE	19.2 ^ef^	4.0 ^fghi^	3.6 ^efg^	31.5 ^ghij^	23.8 ^efgh^	0.6 ^cdefgh^	5.3 ^abcdef^	11.8 ^defg^	63.6 ^ghi^	17.1 ^cdefg^	3.73	1.27
Julius SE	24.1 ^gh^	2.8 ^ab^	3.1 ^cde^	29.7 ^efg^	20.8 ^abcd^	0.5 ^bcdef^	5.8 ^bcdefg^	13.2 ^fghi^	56.8 ^bcd^	19.0 ^fghijk^	2.98	1.34
Kerubino	16.7 ^cde^	4.1 ^ghij^	5.5 ^k^	28.1 ^cde^	23.9 ^efgh^	0.8 ^fghij^	5.6 ^bcdefg^	15.2 ^jklm^	62.4 ^fgh^	20.8 ^klm^	3.01	1.33
Lancer	13.8 ^abc^	3.6 ^cdefg^	4.5 ^hij^	29.0 ^def^	24.6 ^ghij^	0.4 ^abcde^	7.7 ^i^	16.2 ^klmn^	62.1 ^efgh^	23.9 ^no^	2.60	1.38
Lili	21.2 ^fg^	3.5 ^bcdefg^	3.8 ^fgh^	27.3 ^cd^	23.5 ^efgh^	0.6 ^cdefgh^	6.0^defgh^	13.9 ^hij^	58.8 ^bcdef^	19.8 ^hijkl^	2.96	1.34
Mace	15.2 ^bcd^	6.5 ^k^	2.8 ^cd^	29.8 ^efgh^	27.2 ^kl^	0.5 ^bcdef^	7.6 ^i^	10.2 ^cd^	66.8 ^ij^	17.8 ^efghi^	3.76	1.27
Patras	18.9 ^def^	4.9 ^j^	4.2 ^ghi^	30.5 ^fghi^	23.7 ^efgh^	0.8 ^fghij^	5.3 ^abcdef^	11.5 ^def^	64.1 ^ghij^	16.8 ^cdef^	3.82	1.26
Penhold	11.1 ^a^	4.0 ^fghi^	7.0 ^l^	32.3 ^ij^	27.2 ^kl^	0.3 ^abc^	5.6 ^bcdef^	12.3 ^efgh^	70.8 ^lm^	17.9 ^fghij^	3.96	1.25
Quarna	17.4 ^cdef^	4.4 ^hij^	4.7 ^ij^	30.8 ^fghi^	21.7 ^bcde^	0.6 ^cdefgh^	9.2 ^j^	11.1 ^cde^	62.2 ^fgh^	20.2 ^jklm^	3.08	1.32
Revelation	18.6 ^def^	3.5 ^bcdefg^	3.4 ^def^	30.7 ^fghi^	22.7 ^cdefg^	1.0 ^jk^	5.1 ^abcde^	14.9 ^ijk^	61.3 ^efgh^	20.0 ^ijkl^	3.07	1.33
Siskin	18.0 ^def^	4.7 ^ij^	2.8 ^cd^	32.0 ^ij^	26.4 ^ijk^	0.7 ^defghi^	5.9 ^cdefg^	9.5 ^abc^	66.5 ^ij^	15.4 ^bcde^	4.32	1.23
Spitfire	17.2 ^cde^	4.2 ^ghij^	3.4 ^def^	27.5 ^cd^	22.4 ^bcdef^	0.9 ^ghij^	7.7 ^i^	16.5 ^klmn^	58.3 ^bcde^	24.2 ^o^	2.41	1.41
Steller	11.9 ^ab^	6.3 ^k^	7.5 ^l^	27.4 ^cd^	28.9 ^l^	0.6 ^bcdefg^	5.8 ^bcdefg^	11.6 ^def^	70.6 ^klm^	17.4 ^defgh^	4.06	1.25
Suntop	15.6 ^bcde^	2.6 ^a^	4.3 ^hi^	31.3 ^ghij^	25.3 ^hijk^	0.4 ^abcd^	7.6 ^i^	12.9 ^efgh^	63.8 ^ghi^	20.4 ^klm^	3.13	1.32
Zulu	17.8 ^edf^	3.7 ^defgh^	3.6 ^efg^	37.1 ^l^	23.0 ^defg^	0.5 ^bcdef^	4.7 ^ab^	9.5 ^abc^	67.9 ^jkl^	14.2 ^ab^	4.79	1.21
Min	11.1	2.6	1.9	21.9	19.0	0.1	4.3	7.8	48.5	12.0	2.07	1.19
Max	28.7	8.9	9.0	37.1	29.2	1.3	9.6	17.2	73.262.6	26.8	5.34	1.48
Mean	18.3	4.2	4.1	30.0	23.8	0.6	6.2	12.7		18.9	3.46	1.31

Within each column, mean values marked with difference letters are significantly different (*p* < 0.05, one-way ANOVA, Tukey HSD).

**Table 2:** R5 reactivities of isolated total gluten from 27 wheat cultivars and C-hordein reference materials.

Cultivar	EC50, ng/mL	Cultivar	EC50, ng/mL	Calibrant/Reference Material	EC50, ng/mL
Amaretto	38.1	Kerubino	74.8	Average of all cultivars	62.1
Anniina	85.9	Lancer	31.2	10% C-hordein	61.5
Apache	376.6	Liili	61.2	20% C-hordein	37.5
Brandon	35.0	Mace	140	30% C-hordein	27.5
Britannia	664.3	Patras	47.9		
Brons	324.6	Penhold	57.7		
Cellule	57.9	Quarna	51.4		
Claire	38.6	Revelation	53.4		
Crusoe	30.4	Siskin	66.9		
Foremost	142.7	Spitfire	55.0		
Gregory	103.8	Steller	30.7		
Hereford	147.9	Suntop	32.6		
Julius DE	80.9	Zulu	66.8		
Julius SE	40.0				

**Table 3:** R5 reactivity of prolamin types of cv. Crusoe analyzed by sandwich R5 enzyme-linked immunosorbent assay (ELISA) and their EC50 value from a non-linear four-parameter curve fit.

Gluten Type	EC50 (ng/mL)
ω1.2-gliadin	13.7
γ-gliadin	44.3
α-gliadin	35.9
HMW glutenin	91.9
LMW glutenin	473.3
ω5-gliadin	--

**Table 4:** R5 reactivity of gliadins from several wheat cultivars with distinct high performance liquid chromatography (HPLC) chromatogram patterns.

Cultivar	ω1.2-Gliadins (EC50, ng/mL)	Cultivar	α-Gliadins (EC50, ng/mL)	γ-Gliadins (EC50, ng/mL)
Amaretto	10.6	Amaretto	31.9	21.3
Anniina	5.3	Apache	26.7	17.0
Brandon	11.4	Brandon	41.6	33.7
Claire	14.0	Foremost	30.7	22.0
Lili	8.9			

**Table 5:** Calculation of gluten content in spiked oat flour based on fresh weight and three ELISA calibration results.

Wheat Cultivar Used in Spiking	Level of Flour Spiked in Oat Flour (mg/kg)	Protein Content of the Wheat Flour	Proportion of Gluten in Total Protein (From Table 1)	Theoretical Gluten Spiked Level in Oat Flour (mg/kg)	ELISA Calibration Result: Gliadin × 2 (mg/kg)	ELISA Calibration Result: Gliadin × CF (mg/kg)	ELISA Calibration Result: 10% C-Hordein (mg/kg)
Apache	1000	10.2%	71.3%	72.7	35 ± 2	26 ± 2	23 ± 1
Zulu	1000	11.7%	82.2%	96.2	80 ± 5	48 ± 3	50 ± 3
Steller	1000	17.8%	88.1%	156.8	176 ± 3	110 ± 2	107 ± 2

Note: CF, cultivar-specific conversion factor obtained from Table 1.

**Table 6:** Calculation of gluten content in spiked oat biscuits based on fresh weight and three ELISA calibration results.

Wheat Cultivar Used in Spiking	Gluten Content in the Spiked Oat Biscuits Flour (mg/kg)	Gluten Content in the Biscuit Recipe (mg/kg)	Theoretical Gluten Content After Baking Moisture Loss, (mg/kg)	ELISA Calibration Result: Gliadin × 2 (mg/kg)	ELISA Calibration Result: Gliadin × CF (mg/kg)	ELISA Calibration 10% C-Hordein (mg/kg)
Apache	72.7	33.0	37.7	27 ± 2	20 ± 1	17 ± 1
Zulu	96.2	43.7	49.5	36 ± 1	22 ± 1	23 ± 1
Steller	156.8	71.3	80.4	85 ± 4	53 ± 2	52 ± 2

## Supplementary Materials

The following are available online at https://www.mdpi.com/2304-8158/9/11/1637/s1, Figure S1: R5 reactivity of total gluten from 27 wheat cultivars and C-hordein calibrates. The EC50 value was calculated from a non-linear four-parameter curve fit (Graphpad Prism 8). EC50 indicates the protein concentration that provokes half of the antibody response. The solid line indicates the fitted curves, and the dotted lines indicated the 95% of confidence intervals. Figure S2: R5 reactivity of gluten types from wheat cv. Crusoe. The EC50 value was calculated from a non-linear four parameter curve fit (Graphpad Prism 8). EC50 indicates the protein concentration that provokes half of the antibody response. The solid line indicates the fitted line, and the dotted lines indicate the 95% confidence intervals. If there were less than 4 data points then it was not possible calculate the confidence intervals. Figure S3: R5 reactivity of gliadin types from different wheat cultivars. The EC50 value was calculated from a non-linear four parameter curve fit (Graphpad Prism 8). The EC50 indicates the protein concentration that provokes half of the antibody response. The solid line indicates the fitted line, and the dotted lines indicate the 95% confidence intervals. If there were less than 4 data points, then it was not possible calculate the confidence intervals. Figure S4: Correlation of the proportion of ω1,2-gliadin in wheat proteins and the logEC50 value of the total gluten isolate from 27 cultivars. The curve was drawn in power fit.
