# Supplementary material for: Barley C-Hordein as the Calibrant for Wheat Gluten Quantification
Source: Foods. 2020 Nov 10;9(11):1637. doi: 10.3390/foods9111637 (PMC7697280; doi:10.3390/foods9111637)
Supplement: Supplementary file 1 [file foods-09-01637-s001.pdf]

Supplementary materials

Barley C-hordein as the calibrant for wheat gluten quantification

Xin Huang *, Kaiyue Ma, Sara Leinonen and Tuula Sontag-Strohm

Department of Food and Nutrition, Faculty of Agriculture and Forestry, University of Helsinki, Agnes Sjöberginkatu 2, PL66, FI-00014, Helsinki, Finland

* Correspondence: xin.huang@helsinki.fi

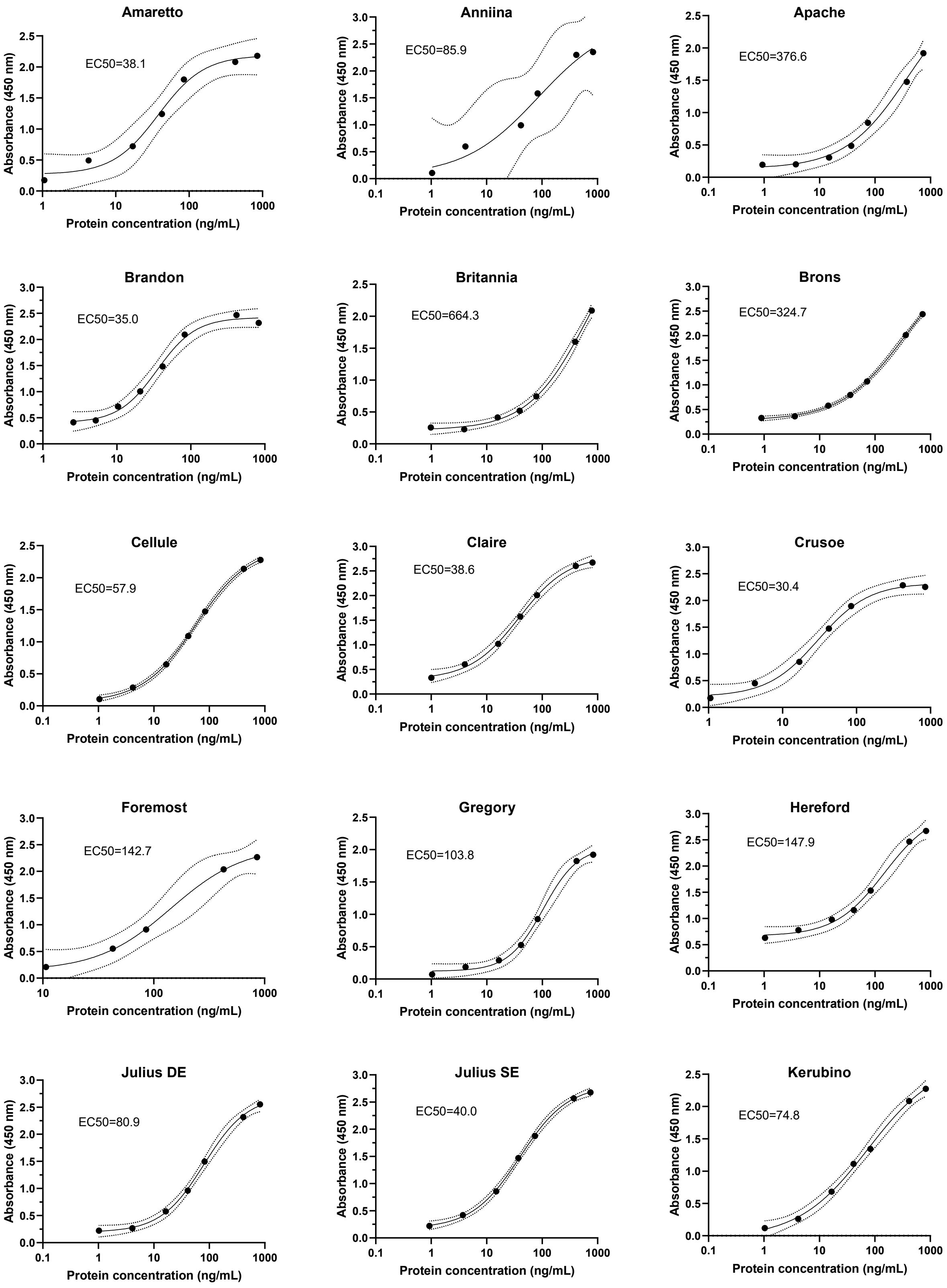
Amaretto
EC50=38.1
Absorbance (450 nm)
Protein concentration (ng/mL)
Anniina
EC50=85.9
Absorbance (450 nm)
Protein concentration (ng/mL)
Apache
EC50=376.6
Absorbance (450 nm)
Protein concentration (ng/mL)
Brandon
EC50=35.0
Absorbance (450 nm)
Protein concentration (ng/mL)
Britannia
EC50=664.3
Absorbance (450 nm)
Protein concentration (ng/mL)
Brons
EC50=324.7
Absorbance (450 nm)
Protein concentration (ng/mL)
Cellule
EC50=57.9
Absorbance (450 nm)
Protein concentration (ng/mL)
Claire
EC50=38.6
Absorbance (450 nm)
Protein concentration (ng/mL)
Crusoe
EC50=30.4
Absorbance (450 nm)
Protein concentration (ng/mL)
Foremost
EC50=142.7
Absorbance (450 nm)
Protein concentration (ng/mL)
Gregory
EC50=103.8
Absorbance (450 nm)
Protein concentration (ng/mL)
Hereford
EC50=147.9
Absorbance (450 nm)
Protein concentration (ng/mL)
Julius DE
EC50=80.9
Absorbance (450 nm)
Protein concentration (ng/mL)
Julius SE
EC50=40.0
Absorbance (450 nm)
Protein concentration (ng/mL)
Kerubino
EC50=74.8
Absorbance (450 nm)
Protein concentration (ng/mL)

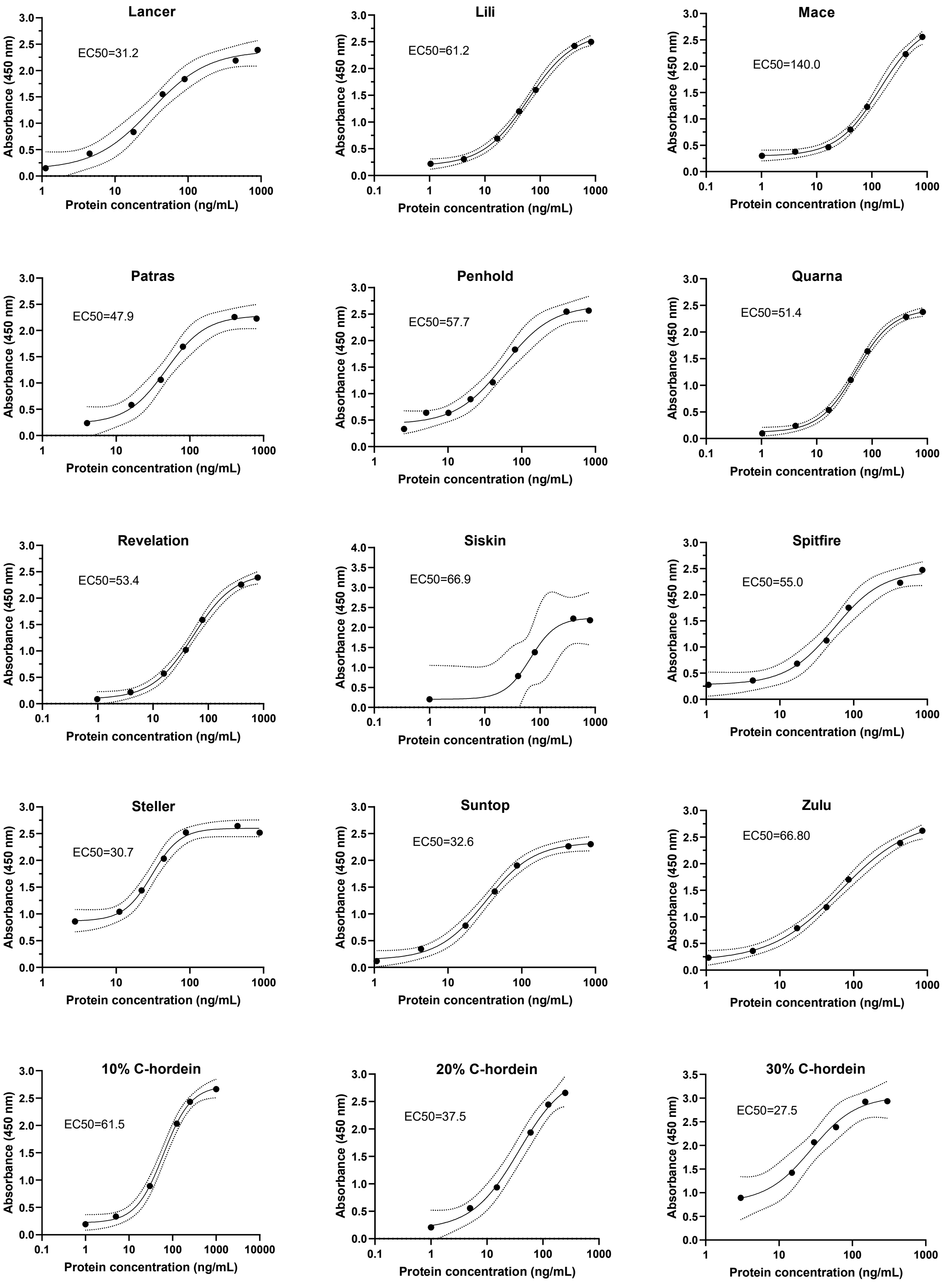

Figure S1. R5 reactivity of total gluten from 27 wheat cultivars and C-hordein calibrates. The EC50 value was calculated from non-liner four parameter curve fit (Graphpad Prism 8). EC50 indicates the protein concentration that provokes half of the antibody response. Solid line indicates the fitted curve, and dotted lines indicate the 95% of confidence intervals.

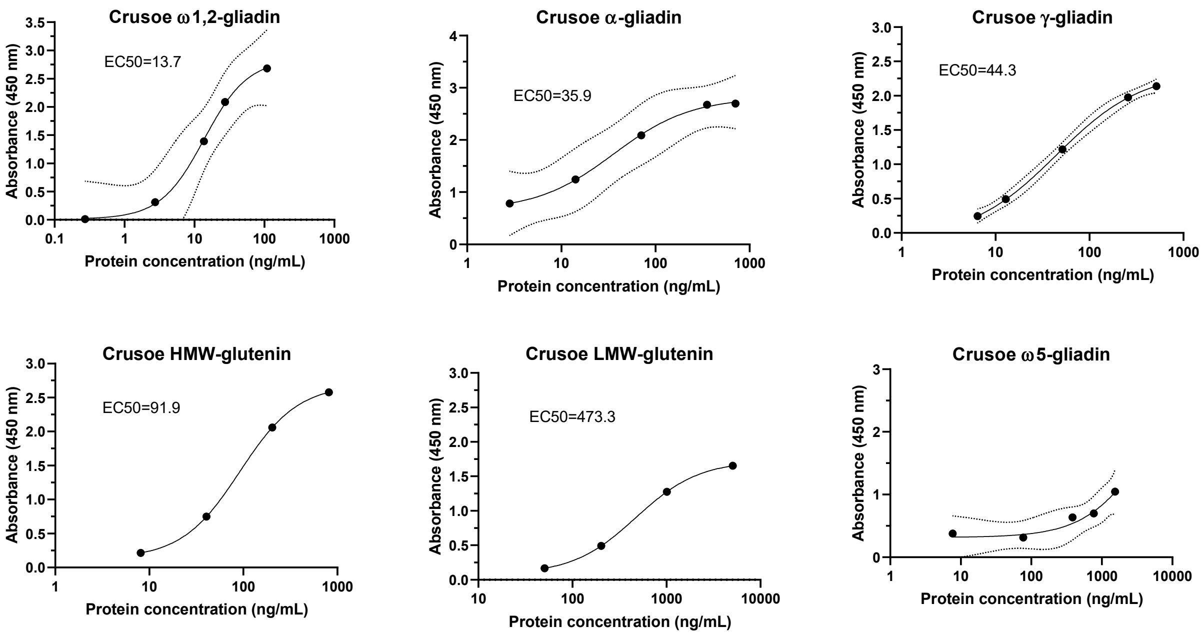

Figure S2. R5 reactivity of gluten types from wheat cv. Crusoe. The EC50 value was calculated from non-liner four parameter curve fit (Graphpad Prism 8). EC50 indicates the protein concentration that provokes half of the antibody response. Solid line indicates the fitted line, and dotted lines indicate the 95% of confidence intervals. Less than 4 data points was not possible calculate the confidence intervals.

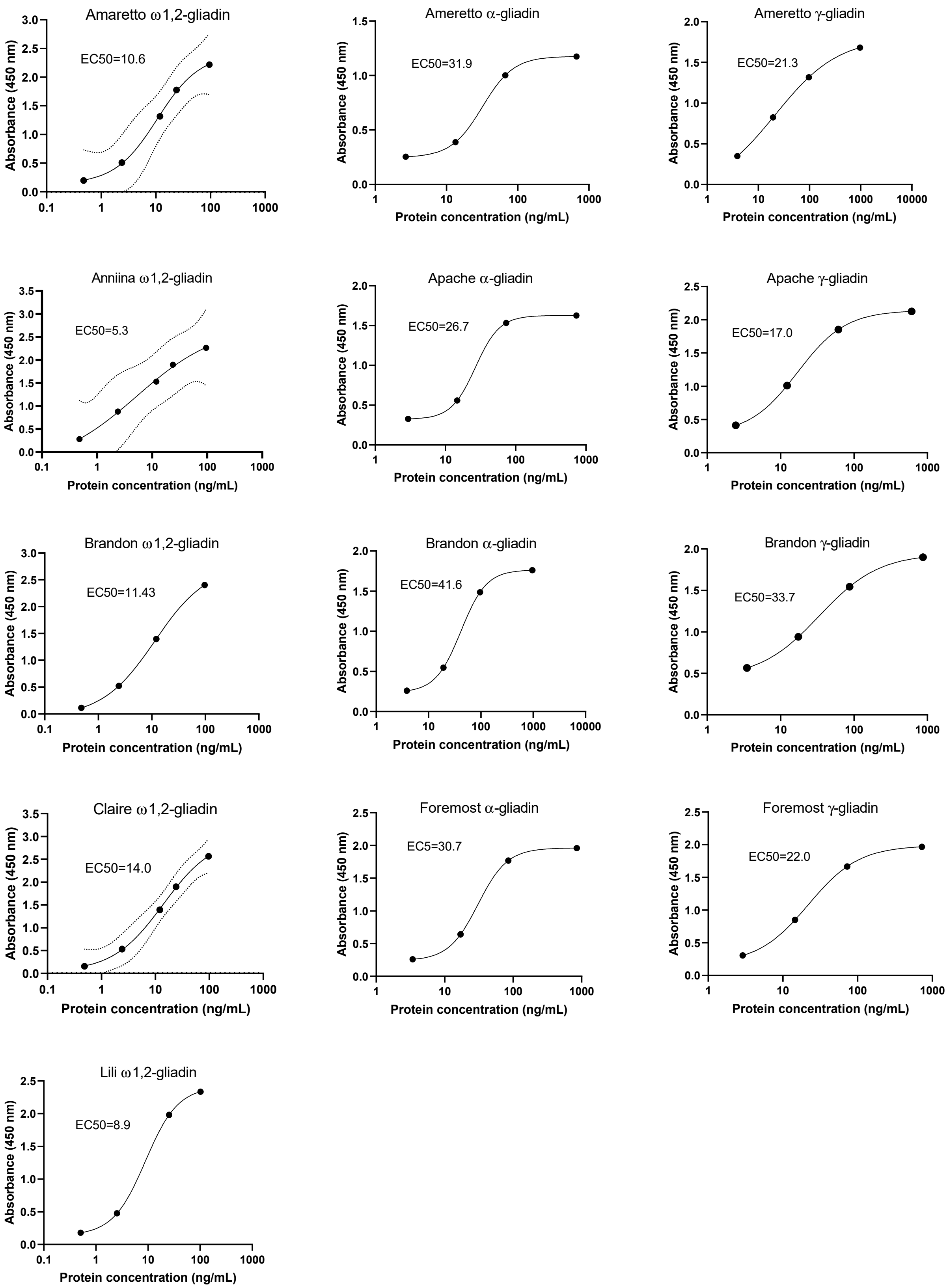

Figure S3. R5 reactivity of gliadin types from different wheat cultivars. The EC50 value was calculated from non-liner four parameter curve fit (Graphpad Prism 8). EC50 indicates the protein concentration that provokes half of the antibody response. Solid line indicates the fitted line, and dotted lines indicate the 95% of confidence intervals. Less than 4 data points was not possible calculate the confidence intervals.

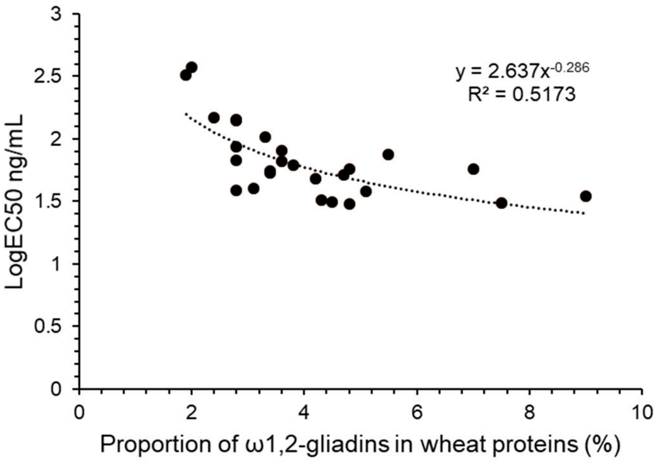

Figure S4. Correlation of the proportion of ω1,2-gliadin in wheat proteins and logEC50 value of the total gluten isolate from 27 cultivars. The curve was in power fit.
